# Determinants of use and non-use of a web-based communication system in cerebral palsy care: evaluating the association between professionals' system use and their *a priori *expectancies and background

**DOI:** 10.1186/1472-6947-11-43

**Published:** 2011-06-18

**Authors:** Jitske Gulmans, Miriam MR Vollenbroek-Hutten, Lisette JEWC van Gemert-Pijnen, Wim H van Harten

**Affiliations:** 1 Roessingh Research & Development, Research Institute for Rehabilitation and Technology, PO Box 310, 7500 AH Enschede, The Netherlands; 2 University of Twente, Faculty of Behavioural Sciences, Department of Psychology Health & Technology, PO Box 217, 7500 AE Enschede, The Netherlands; 3 University of Twente, School of Management and Governance, Department of Health Technology and Services Research, PO Box 217, 7500 AE Enschede, The Netherlands

## Abstract

**Background:**

Previously we described parents' and professionals' experiences with a web-based communication system in a 6-month pilot in three Dutch cerebral palsy care settings. We found that half of the participating professionals had not used the system, and of those who had used the system one third had used it only once. The present study aimed to evaluate whether professionals' system use was associated with their *a priori* expectancies and background.

**Methods:**

Professionals who had not used the system (n = 54) were compared with professionals who had used the system more than once (n = 46) on the basis of their questionnaire responses before the pilot, their affiliation and the number of patients which they represented in the study. The questionnaire items comprised professionals' expectancies regarding the system's performance and ease of use, as well as the expected time availability and integration into daily care practice.

**Results:**

Overall, users had higher *a priori *expectancies than non-users. System use was associated with expected ease of use (p = .046) and time availability (p = .005): 50% of the users (vs. 31% of the non-users) expected that the system would be easy to use and 93% of the users (vs. 72% of the non-users) expected that they would be able to reserve a time slot each week for responding to submitted questions. With respect to professionals' affiliation, system use was associated with professionals' institution (p = .003) and discipline (p = .001), with more (para-) medical professionals among users (93% vs. 63% among non-users), and more education professionals among non-users (37% vs. 7% among users). In addition, users represented more patients (mean 2, range 1-8) than non-users (mean 1.1, range 1-2) (p = .000).

**Conclusions:**

Professionals' system use was associated with expected ease of use and time availability, professionals' affiliation and the number of represented patients, while no association was found with expected performance of the system. To achieve higher adoption rates in the future, it is important to further develop the technology by optimizing the system's ease of use and interoperability and including advanced consultation options. In addition, better identified end users should be more extensively informed about the system's possibilities through tailored education.

## Background

Children with special health care needs (CSHCN) are an important population from health care services, economic and policy perspectives [1]. The CSHCN population involves 'children who have or are at increased risk for a chronic physical, developmental, behavioural, or emotional condition and who also require health and related services of a type or amount beyond that required by children generally' [2]. The broad range of care needs in this population often requires complex and long-term health (related) services from multiple providers across diverse organisations and sectors. In such 'integrated care' settings, inter-professional communication about children's needs, family context, and prior experiences with and responses to health care is essential for effective coordination of services [3]. However, the findings of the U.S. National Survey of CSHCN 2005-2006 revealed that those children most in need of comprehensive, coordinated systems of care were the least likely to receive such care [4]. In addition, a study among CSHCN populations with neurological conditions found that children with multiple conditions had the greatest unmet needs and dissatisfaction with care coordination [5], which was defined in terms of communication among doctors and between doctors and other providers and whether the family received sufficient help coordinating care, if needed. Failure of professionals caring for the same child to communicate with one another often leaves the parents as information intermediaries [6]. This corresponds to our findings on the care of children with cerebral palsy in the Netherlands (see appendix 1), in which we identified various gaps in patient care communication, such as lack of patient centredness and poor inter-professional information exchange, leading to parents acting as messengers of information, as well as to hesitation among professionals to contact each other due to unfamiliarity with those involved in the care network [7].

Although much has been written about the potential of telemedicine to increase access to care, applications in paediatrics are relatively scarce [8]. Nevertheless, they are increasingly being applied to facilitate communication between health care providers and caregivers of paediatric patients with health conditions requiring follow-up [9,10]. Examples include applications using synchronous video-conferencing as the most common mode of communication, and consultation and diagnosis as the most common function [9]. In order to improve parent-professional and inter-professional communication in three Dutch cerebral palsy care settings, we developed an asynchronous, secure web-based communication system aimed at increasing patient centeredness, facilitating inter-professional contact and enhancing network transparency. Previously we described its design features, technical feasibility and clinical usability with respect to its aims, as well as parents' and professionals' actual system use in a 6-months pilot in three Dutch care regions. We found that half of the participating professionals had not used the system at all and of the professionals who had used the system, a third had used it only once [11]. To enable the development of services with a higher adoption rate it is important to obtain insight into the determinants of use and non-use [12], which might facilitate the definition of user requirements and hence a better fit between user requirements and the system. Functional user requirements generally concern the clinical value/targeted performance, while non-functional requirements mostly concern ease of use, both of which are considered important determinants of usage intention and subsequent usage behaviour [13,14]. The aim of this study was therefore to evaluate whether professionals' *a priori *expectancies regarding the system's performance and ease of use were associated with their subsequent use and non-use of the system. In addition, as focus groups convened in the development phase of the project revealed the importance of time availability and integration into daily practice and the role of professional background, these aspects were evaluated as well. The evaluation was performed on user level, comparing professionals who had not used the system (n = 54) with professionals who had used the system more than once (n = 46), hypothesizing higher *a priori *expectancies in the use-group. Professional background was evaluated in terms of professionals' affiliation (care region, institution, discipline) and the number of patients which they represented in the study, hypothesizing that professionals in the use-group would represent a higher number of patients.

### Appendix 1) Cerebral palsy care in the Netherlands

In the Netherlands, paediatric rehabilitation services are delivered in both inpatient and outpatient settings. The 23 national rehabilitation centres with paediatric facilities and the rehabilitation departments of all medium-sized and larger hospitals offer treatment on an outpatient basis only. For inpatient treatment children can be referred to one of nine specialized, regional rehabilitation centres. Annually, 6,755 children are treated on an outpatient basis and 363 children on an inpatient basis [15]. More than half of these children have been diagnosed with cerebral palsy, an umbrella term for a group of motor disorders which cause activity limitation and are often accompanied by disturbances of sensation, perception, cognition, communication and behaviour, by epilepsy, and by secondary musculoskeletal problems. While its prevalence ranges from 1.5 to 2.5 per 1000 live births with little or no variation among western nations, the importance of cerebral palsy is particularly related to its severity and its consequent burden on affected children, families and societies [16]. Given the broad range of disabilities associated with cerebral palsy, various professionals from diverse organizations are involved in meeting each patient's care needs. In the Netherlands, cerebral palsy patients aged 4-8 years usually are under the supervision of a rehabilitation physician in a (specialized) regional or academic hospital, who often plays a coordinating role in the integral medical care. At the age of 4, the children are referred either to regular schools (whether or not assisted by ambulant supervision) or to schools for special education/specialized day care centres. Children in regular education can often do with outpatient supervision combined with mono-disciplinary therapy in a primary care centre. Schools for special education usually have close cooperation with the local rehabilitation centre, while specialized day care centres are often supported by ambulant consultation of a rehabilitation physician.

## Methods

### Study population

To obtain data representative for the integrated care setting of cerebral palsy, the study covered three Dutch care regions ranging from urban to more rural settings. The inclusion of professionals was based on the inclusion of cerebral palsy patients and their parents, which was determined by a rehabilitation physician based on specific selection criteria which we described in our previous study [11]. Of all the professionals involved in the care of the 30 selected cerebral palsy patients, 120 (67%) were willing to participate in the study. Both parents and professionals gave informed consent after which they received log-in details for system access. System use was on a voluntary basis, i.e. professionals were free in their choice to use the system in a given situation or to apply their usual modes of communication (face-to-face, telephone etc.). The study was conducted in keeping with the protocol of the WMA Declaration of Helsinki. According to Dutch legislation (WMO Medical Research Involving Human Subjects Act) a medical ethics review was not required.

### System use and non-use

The system comprised an open access part (a generally accessible website with project related information) and a personalized secured access part with various consultation options. Professionals could contact the parents of the patient(s) whom they represented in the study, as well as colleague-professionals involved in the patient's care network. A detailed description of the system's technical and functional specifications can be found in our previous study [11]. Of the 120 participating professionals, 54 had not used the system during the 6-month pilot (of which 33 did log into the system), 20 professionals had used the system only once and 46 had used it more than once (with a mean of n = 6 questions/responses per professional, sd 5 and range 2-23). In view of the system's aim to facilitate inter-professional contact, professionals' system use was defined in terms of submitting a question/response in the system more than once. Consequently, trying out the system only once or logging-in without submitting a question/response was not considered actual system use. The definition of use in terms of using the system more than once was made quite arbitrarily, but with the intention to create a real contrast between the use and non-use group.

### Study design

We verified that all 120 participating professionals completed the baseline questionnaire before obtaining access to the web-based system. In table 1 an overview is given of the questionnaire items. Performance expectancy items were derived from the system's aims, which were based on the experienced gaps in communication we previously identified in cerebral palsy care settings [7]. For the majority of items a 5-point Likert-scale was used, ranging from *'no, not at all' *to *'yes, definitely' *with open text area for clarification. The items on inter-professional communication (i.e. *frequency of contact, accessibility of professionals *and *facilitation of inter-professional consultation*) were assessed by means of a scoring table in which professionals could mark their response for each professional involved in their care network *('rarely'/'occasionally'/'regularly' *for the item *frequency of contact *and *'yes'/'don't know'/'no' *for the items *accessibility of professionals *and *facilitation of inter-professional consultation*).

**Table 1:** Questionnaire items evaluating professionals' a priori expectancies of the web-based system.

Performance	*Frequency of contact* How often do you expect to contact colleague-professionals through use of the system?
	*Accessibility of professionals* Do you expect that you will be able to reach colleague-professionals more easily through use of the system?
	*Facilitation of inter-professional consultation* Do you expect that you will be able to consult colleague-professionals more often through use of the system?
	*Parents' messenger role* Do you expect that as a result of using the system parents less often have to act as messenger of information between professionals?
	*Consistency of information* Do you expect that as a result of using the system more consistent information can be given to parents?
	*Transparency of care network* Do you expect that as a result of using the system the child's care network will be more transparent? (i.e. who is involved from which organization etc.)
**Ease of use**	Do you expect that the system will be easy to use for you?

**Time availability**	Do you expect that you will be able to reserve one time slot each week for responding to submitted questions on the system?

**Integration daily care practice**	Do you expect that you will be able to reserve time for system use within your regular working hours?

The individual scores in each scoring table were recoded into one total score. For the item *frequency of contact *the total score was *'regularly' *when the respondent expected to have regular contact with at least one of his/her colleague-professionals. When this was not the case, the total score was *'occasionally' *when the respondent expected to have occasional contact with at least one of his/her colleague-professionals. When the respondent did not expect to have regular nor occasional contact with any of his/her colleague-professionals, the total score was *'rarely'*.

For the items *accessibility of professionals *and *facilitation of inter-professional consultation *a positive total score (*'yes'*) was given when the respondent expected to resp. reach/consult at least one of his/her colleague-professionals more easily/more often. A negative total score (*'no'*) was given when the respondent did not expect to reach/consult any of his/her colleague-professionals more easily/more often. A neutral total score was assigned when the respondent did not know what to expect.

### Data analysis

Professionals who had not used the system (n = 54) were compared with professionals who had used the system more than once (n = 46). Professionals who had used the system only once (n = 20) were included in descriptive overviews, but were left out of the statistical analysis in order to create a real contrast between the use and non-use group. For the comparison of professionals' expectancies, Fisher Exact tests (α = .05, df = 1) were applied, contrasting the upper response category (positive expectancy scores) with the latter two response categories (neutral and negative expectancy scores), using one-sided p-values in line with our hypothesis that users would have higher expectancies than non-users. For the comparison of professionals' affiliation and the number of patients which professionals represented in the study, Pearson Chi-square tests were applied (α = .05, 2-sided). Given the skewed distribution of the number of represented patients (the majority of professionals participated in the study for only 1 child), it was categorized into an ordinal variable (n = 1, n = 2 and N ≥ 3).

## Results

### System use & professionals' a priori expectancies

In table 2 an overview is given of professionals' *a priori *expectancies in the use and non-use group. As can be seen, both users and non-users had rather high *a priori *expectancies of the system, although expectancies of users were mostly higher than those of non-users. A statistically significant association was found between system use and expected ease of use (p = .046) and time availability (p = .005): 50% of the users vs. 31% of the non-users expected that the system would be easy to use while 93% of the users vs. 72% of the non-users expected that they would be able to reserve one time slot each week for responding to submitted questions. Although no statistically significant association was found between system use and professionals' expectancies regarding the system's performance, users tended to score higher than non-users.

**Table 2:** Overview of professionals' a priori expectancies of the system in the use and non-use group.

				TOTAL (n = 120)		NON-USE (n = 54)		USE = 1 (n = 20)		USE (n = 46)		F. Exact (1-sided)
				N	%	N	%	N	%	N	%	
**Performance**	Frequency of contact	How often do expect to contact colleague-professionals through use of the system?	*regularly*	59	49%	21	39%	15	75%	23	50%	.181
			*occasionally*	58	48%	32	59%	4	20%	22	48%	
			*rarely*	3	3%	1	2%	1	5%	1	2%	
	Accessibility of professionals	Do you expect that you would be able to reach colleague-professionals more easily through use of the system?	*yes*	93	78%	39	72%	15	75%	39	85%	.117
			*don't know*	10	8%	4	7%	4	20%	2	4%	
			*no*	14	12%	9	17%	1	5%	4	9%	
			missing value	3	3%	2	4%	0	0%	1	2%	
	Facilitation of inter-prof. consultation	Do you expect that you would be able to consult colleague-professionals more often through use of the system?	*yes*	84	70%	37	69%	14	70%	33	72%	.316
			*don't know*	12	10%	5	9%	4	20%	3	7%	
			*no*	21	18%	12	22%	1	5%	8	17%	
			missing value	3	3%	0	0%	1	5%	2	4%	
	Parents' messenger role	Do you expect that as a result of using the system parents less often have to act as messenger of information between professionals?	*yes (definitely)*	84	70%	35	65%	16	80%	33	72%	.245
			*don't know*	30	25%	17	31%	4	20%	9	20%	
			*no (not at all)*	5	4%	2	4%	0	0%	3	7%	
			missing value	1	1%	0	0%	0	0%	1	2%	
	Consistency of information	Do you expect that as a result of using the system, more consistent information can be given to parents?	*yes (definitely)*	72	60%	30	56%	12	60%	30	65%	.179
			*don't know*	40	33%	19	35%	7	35%	14	30%	
			*no (not at all)*	7	6%	5	9%	1	5%	1	2%	
			missing value	1	1%	0	0%	0	0%	1	2%	
	Transparency of care network	Do you expect that as a result of using the system the child's care network will be more transparent?	*yes (definitely)*	65	54%	31	57%	10	50%	24	52%	.468
			*don't know*	47	39%	19	35%	10	50%	18	39%	
			*no (not at all)*	6	5%	4	7%	0	0%	2	4%	
			missing value	2	2%	0	0%	0	0%	2	4%	

**Ease of use**		Do you expect that the system will be easy to use for you?	*yes (definitely)*	48	40%	17	31%	8	40%	23	50%	.046*
			*don't know*	71	59%	37	69%	12	60%	22	48%	
			*no (not at all)*	1	1%	0	0%	0	0%	1	2%	

**Time availability**		Do you expect that you will be able to reserve one time slot each week for responding to questions on the system?	*yes (definitely)*	99	83%	39	72%	17	85%	43	93%	.005*
			*don't know*	17	14%	11	20%	3	15%	3	7%	
			*no (not at all)*	4	3%	4	7%	0	0%	0	0%	

**Integration daily care practice**		Do you expect that you will be able to reserve time for system use within your regular working hours?	*yes (definitely)*	65	54%	28	52%	12	60%	25	54%	.514
			*don't know*	19	16%	6	11%	5	25%	8	17%	
			*no (not at all)*	33	28%	18	33%	3	15%	12	26%	
			missing value	3	3%	2	4%	0	0%	1	2%	

### System use & professional background

In table 3 an overview is given of professionals' affiliation (care region, institution, discipline) and the number of represented patients in the use and non-use group. With respect to the professional's care region, the use-group had more professionals from the rural care region C (50% vs. 30% in the non-use group), while the non-use group had more professionals from the urban care region A (39% vs. 22% in the use-group), although this was not a statistically significant association. Comparing the professionals' institution, the use-group had more professionals from rehabilitation centres (39% vs. 13% in the non-use group), whereas the non-use group had more professionals from (special) education/day care centres (56% vs. 24% in the use-group), resulting in a significant association between system use and professionals' institution (p = .003). In addition, system use was associated with professionals' discipline (p = .001): the use-group had more (para-) medical professionals (93% vs. 63% in the non-use group) while the non-use group had more education professionals (37% vs. 7% in the use-group). Comparing the number of patients which professionals represented in the study, users represented more patients (mean 2.0, range 1-8) compared to non-users (mean 1.1, range 1-2) (p = .000).

**Table 3:** Overview of professionals' affiliation and number of represented patients in the use and non-use group.

			TOTAL (n = 120)		NON-USE (n = 54)		USE = 1 (n = 20)		USE (n = 46)		X^2^ (2-sided)
			N	%	N	%	N	%	N	%	
**Care region**		region A (urban)	35	29%	21	39%	4	20%	10	22%	.079
		region B (urban/rural)	36	30%	17	31%	6	30%	13	28%	
		region C (rural)	49	41%	16	30%	10	50%	23	50%	

**Institution**		hospital	22	18%	12	22%	0	0%	10	22%	.003*
		rehabilitation centre	30	25%	7	13%	5	25%	18	39%	
		(special) education/day care centre	55	46%	30	56%	14	70%	11	24%	
		primary care centre	13	11%	5	9%	1	5%	7	15%	

**Discipline**	total	medical	23	19%	8	15%	0	0%	15	33%	.001*
		paramedical	66	55%	26	48%	12	60%	28	61%	
		educational	31	26%	20	37%	8	40%	3	7%	
	medical	rehabilitation physician	11	9%	1	2%	0	0%	10	22%	
		paediatrician	8	7%	5	9%	0	0%	3	7%	
		paediatric neurologist	2	2%	1	2%	0	0%	1	2%	
		orthopaedic surgeon	1	1%	1	2%	0	0%	0	0%	
		other	1	1%	0	0%	0	0%	1	2%	
	paramedical	physiotherapist	31	26%	13	24%	3	15%	15	33%	
		occupational therapist	9	8%	1	2%	3	15%	5	11%	
		speech therapist	6	5%	3	6%	2	10%	1	2%	
		manufacturer rehabilitation aids	4	3%	1	2%	1	5%	2	4%	
		pedagogue	5	4%	3	6%	1	5%	1	2%	
		social work	2	2%	0	0%	1	5%	1	2%	
		orthoptist	4	3%	2	4%	0	0%	2	4%	
		other	5	4%	3	6%	1	5%	1	2%	
	educational	teacher	10	8%	6	11%	3	15%	1	2%	
		(ambulant) supervisor	16	13%	11	20%	4	20%	1	2%	
		group leader (day care)	4	3%	3	6%	0	0%	1	2%	
		other	1	1%	0	0%	1	5%	0	0%	

**Npatients**		N_patients _= 1	94	78%	50	93%	16	80%	28	61%	.000*
		N_patients _= 2	14	12%	4	7%	2	10%	8	17%	
		N_patients _≥ 3	12	10%	0	0%	2	10%	10	22%	

## Discussion

The aim of this study was to evaluate whether professionals' use and non-use of a web-based communication system in cerebral palsy care was associated with their *a priori *expectancies and background. Overall, users had higher *a priori *expectancies than non-users. System use was associated with expected ease of use and time availability, while no association was found with professionals' *a priori *expectancies regarding the system's performance. The association with expected ease of use confirms our hypothesis and is conform adoption literature [13,14]. The association with expected time availability is in line with findings in literature reporting providers' concerns that web-based communication would add to their work-load rather than substitute for other tasks [17,18]. Considering the rather high performance expectancies of both users and non-users, one could argue there was little doubt in either of the groups about the expected clinical value of the system, whereas the groups differed in the amount of effort they expected to invest in using the system. Those expecting to have more time available and/or that the system would be easy to use indeed used the system more often. Comparing these findings with frequently used IT adoption models such as the Unified Theory of Acceptance and Use of Technology [14], the lack of association between system use and professionals' expectancies regarding the system's performance is surprising, as performance expectancy is considered a direct determinant of usage intention and subsequent usage behaviour [14]. The fact that we did not find an association might be related to our operationalization of performance in terms of the aims of the web-based system. Although these system aims were derived from experienced gaps in communication previously identified in cerebral palsy care settings [7], an operationalization in broader terms (i.e. system use would improve my job performance/increase my productivity/make it easier to do my job/etcetera [14]) might have better addressed the wide range of professionals' outcome expectancies.

With respect to professionals' affiliation, system use was associated with institution and discipline, with more (para-)medical professionals among users and more education professionals among non-users. On the one hand this could imply that the system was of less use to education professionals: their communication with parents usually comprises face-to-face contact, while their inter-professional communication might be less focused on the integrated care network but more on the internal contact within the school/day care centre. On the other hand, they could have had the intention to use the system, but might not have needed to use it for the particular child they represented in the study, a hypothesis strengthened by the fact that all 31 education professionals who participated did so for only one patient. Indeed, system use was significantly associated with the number of patients which professionals represented in the study: of the professionals who had not used the system the far majority (93%) represented only one patient.

Although professionals' system use was associated with their *a priori *expectancies and background, the differences between users and non-users were not as pronounced as might be expected. From a methodological point of view, this might be related to the fact that professionals who had not used the system at all were compared with professionals who had used the system more than once. This low cut-off point was chosen given the limited range of frequency of use. A clearer contrast between use and non-use and larger population series might have yielded more pronounced differences between both groups.

The evaluation in the present study was performed on the level of individual users and was not focused on the inter-professional and inter-organizational environment that is inherent to integrated care settings such as cerebral palsy. Adoption of innovative technologies that span professional and institutional boundaries pose challenges in terms of coordination of care processes, such as changing handovers, alignment of objectives and working culture and integrating the technology in each different setting [19]. To ensure that health care technologies are effectively used, an approach is needed that incorporates the complex interdependencies between technology, people and their social-cultural environment [20]. Usually the design and pilot evaluation phases require an interactive process of co-creation and close collaboration with intended users and stakeholders [20], and it will take a while before the technology is sufficiently stable for broad diffusion and interoperable across organizational and social contexts and technical infrastructures [21]. These dynamics are to be taken into account when deciding on an evaluation method. In order to generate usable evidence in the early stages of the fast changing field of telehealth [22], new methodologies such as Constructive Technology Assessment [23] or a holistic approach for the design and evaluation of eHealth technologies [20] can be considered.

## Conclusions

For a better understanding of the adoption of telemedicine applications, analysis of determinants of use and non-use is essential. The findings of the present study suggest that users and non-users differ from each other with respect to some of their *a priori *expectancies, their affiliation and the number of patients which they represented in the study. This information can be taken into account in the further implementation of the system in every day care, but also by making system adaptations in order to increase the chance of professionals' system use. Considering the users' higher expectancies of the system's ease of use, this aspect could be further optimized by reducing the amount of time involved in system use and providing a better integration of the system in daily care practice by linking the system's communication automatically with existing patient records. As performance expectancies are generally considered a strong determinant of system use, tailored education addressing the broad range of professionals' outcome expectancies may contribute to adoption. With respect to professional background, system use by education professionals might be stimulated through advanced consultation options tailored to their specific needs, provided that the number of patients for which they participate is large enough in order to adequately engage in the system.

In line with a staged approach to telemedicine evaluation, the present study had an explorative character and focused on a limited number of factors that could explain professionals' system use and non-use. Further research may include a more comprehensive evaluation of technology, human and organization issues, in which multivariate analysis can be used to gain insight into the relative contribution of these factors.

## Competing interests

The authors declare that they have no competing interests.

## Authors' contributions

As project-leader JG was involved in all phases of the study. MVH, JGP and WH participated in its design and coordination and helped to draft the manuscript. All authors read and approved the final manuscript.

## Acknowledgements and funding

We especially want to thank Jules Becher (VU University Medical Centre), Karel Maathuis (University Medical Centre Groningen), Cathrien van Groningen (Roessingh Rehabilitation Centre) and Jacqueline Visser (Roessingh Research and Development) for their contribution to this study. We gratefully acknowledge the Johanna Child Fund, the Child Fund Adriaanstichting and the National Innovation Centre for Rehabilitation Technology for funding.

## Pre-publication history

The pre-publication history for this paper can be accessed here:

http://www.biomedcentral.com/1472-6947/11/43/prepub
